# Microbiome dynamics of human epidermis following skin barrier disruption

**DOI:** 10.1186/gb-2012-13-11-r101

**Published:** 2012-11-15

**Authors:** Patrick LJM Zeeuwen, Jos Boekhorst, Ellen H van den Bogaard, Heleen D de Koning, Peter MC van de Kerkhof, Delphine M Saulnier, Iris I van Swam, Sacha AFT van Hijum, Michiel Kleerebezem, Joost Schalkwijk, Harro M Timmerman

**Affiliations:** 1 Nijmegen Centre for Molecular Life Sciences (NCMLS), Radboud University Nijmegen Medical Centre, PO BOX 9101, 6500 HB Nijmegen, The Netherlands; 2 Nijmegen Institute for Infection, Inflammation and Immunity (N4i), Radboud University Nijmegen Medical Centre, PO BOX 9101, 6500 HB Nijmegen, The Netherlands; 3 Centre for Molecular and Biomolecular Informatics (CMBI), Radboud University Nijmegen Medical Centre, PO BOX 9101, 6500 HB Nijmegen, The Netherlands; 4 NIZO Food Research B.V., Kernhemseweg 2, 6718 ZB, Ede, The Netherlands; 5 Wageningen University, Host-Microbe Interactomics Group, De Elst 1, 6708 WD, Wageningen, The Netherlands

## Abstract

**Background:**

Recent advances in sequencing technologies have enabled metagenomic analyses of many human body sites. Several studies have catalogued the composition of bacterial communities of the surface of human skin, mostly under static conditions in healthy volunteers. Skin injury will disturb the cutaneous homeostasis of the host tissue and its commensal microbiota, but the dynamics of this process have not been studied before. Here we analyzed the microbiota of the surface layer and the deeper layers of the stratum corneum of normal skin, and we investigated the dynamics of recolonization of skin microbiota following skin barrier disruption by tape stripping as a model of superficial injury.

**Results:**

We observed gender differences in microbiota composition and showed that bacteria are not uniformly distributed in the stratum corneum. Phylogenetic distance analysis was employed to follow microbiota development during recolonization of injured skin. Surprisingly, the developing neo-microbiome at day 14 was more similar to that of the deeper stratum corneum layers than to the initial surface microbiome. In addition, we also observed variation in the host response towards superficial injury as assessed by the induction of antimicrobial protein expression in epidermal keratinocytes.

**Conclusions:**

We suggest that the microbiome of the deeper layers, rather than that of the superficial skin layer, may be regarded as the host indigenous microbiome. Characterization of the skin microbiome under dynamic conditions, and the ensuing response of the microbial community and host tissue, will shed further light on the complex interaction between resident bacteria and epidermis.

## Background

The microbial diversity of human microbiota is determined by various factors, such as transmission of non-resident microbes, genetic predisposition, host demographic characteristics, lifestyle and environmental characteristics [1,2]. Humans have a complex interaction with resident microbes as they help us to digest food, and keep us healthy by competing with pathogens and educating our immune system [3,4]. The US National Institute of Health-funded Human Microbiome Project Consortium started 5 years ago to characterize the human microbial communities present at specific body sites, including skin [5,6]. These efforts have recently resulted in an extensive map of the microbes that live in and on us [7,8]. Aberrant microbial compositions have been linked to inflammation-associated human diseases, including specific skin diseases like psoriasis, atopic dermatitis, acne, and chronic skin ulcers [9]. Skin injury occurs frequently and is likely to have an impact on the skin microbiota. As skin is relatively easily accessible, and invasive procedures to study skin injury in human subjects are available [10,11], we here studied the dynamics of the cutaneous microbiome in a model for standardized skin barrier disruption.

Recently, advanced molecular analyses of skin microbiota have revealed a considerably greater diversity of organisms than presumed from culture-based methods [12,13]. These studies, using either conventional clone sequencing or next-generation sequencing techniques [14], reported that bacterial diversity mainly depends on the topographical location on the body, and that the observed (minimal) temporal variability of the skin microbiome appeared to be dependent on the site sampled [15-19]. In general, it was demonstrated that our skin microbiome has a high degree of interpersonal variation with a site-specific composition, but the intra-individual variability of the skin microbiota was reported to be lower when sites with bilateral symmetry were compared [15,17,18]. These large scale studies of the composition of microbial communities in healthy volunteers have revealed that most of the resident skin bacteria are categorized into four different phyla: Actinobacteria (most dominant reported genera: *Propionibacterium *and *Corynebacterium*), Firmicutes (majorly represented by *Staphylococcus *spp.), Proteobacteria and Bacteroidetes. In addition to its effect on the host, human skin microflora controls colonization by potentially pathogenic microorganisms [20-23], emphasizing the importance of the human skin microbiome in health and disease [24].

Many human microbiome studies have documented the normal human microbiota composition in a variety of niches and tissues, such as gut, oral cavity, vagina and skin to create a catalogue of resident bacteria using cultivation-independent methods [15,18,25-28]. In addition, disease state or microbiome recovery following antibiotics therapy have been studied to obtain insight into dynamic and pathological situations [29-34]. Sequencing-based microbiome studies of diseased skin are currently limited to psoriasis [35], atopic dermatitis [36], acne vulgaris [37] and chronic diabetic wounds [38]. Using conventional clone sequencing, it was found that the bacterial diversity observed in lesional psoriatic skin was greater than for skin from healthy individuals or non-lesional skin from psoriatic patients. The most abundant and diverse phylum populating the psoriatic lesions was Firmicutes, whereas the phylum Actinobacteria was significantly underrepresented compared to non-lesional skin samples from both healthy persons and patients with psoriasis [35]. It is, however, unclear if disease-associated changes in the microbiota composition have a causal role or are merely the result of abnormal skin biology observed in inflammatory skin diseases like psoriasis and atopic dermatitis [39]. In cases where metagenomic studies of the skin microbiome identify (groups of) microorganisms that are causative, or at least involved in the pathogenesis of diseases, then these will be potential targets for novel therapies.

In the present study we investigated whether skin barrier disturbance or skin barrier repair responses affect the host microbiome. Furthermore, we investigated how microbiota composition differed between layers of the stratum corneum and how these relate to recolonization patterns observed after its removal by tape stripping. To this end, we have used barcoded 454-pyrosequencing of the V3-V4 region of the 16S rRNA gene for in-depth analysis of microbiota composition of all samples. This study reveals that bacteria are not uniformly distributed in the stratum corneum. Clear gender differences were identified in upper buttock skin microbiota composition and in the extent of microbiota disturbances after tape stripping. Furthermore, we identified a consistent pattern in microbial community shifts after injury. Our data suggest that the microbiome of the deeper layer of the stratum corneum has an important role in the recolonization process of injured skin.

## Results

### High bacterial diversity in upper buttock skin

**Figure 1:**
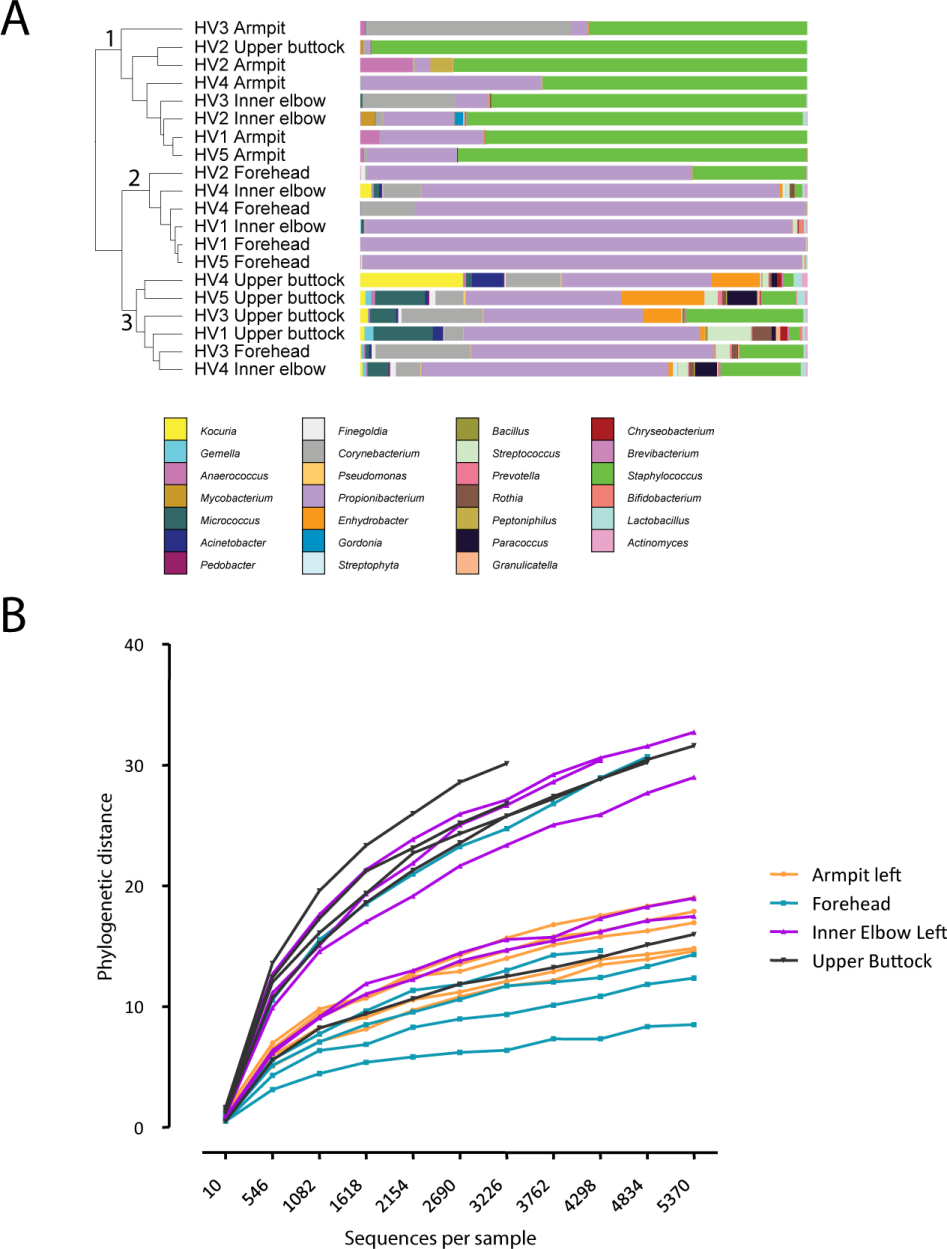
**Clustering, microbial community composition and microbial diversity of samples from different sampling sites**. Composition is displayed as relative abundance, that is, the number of reads assigned to a genus divided by the total number of reads assigned up to the genus level. **(a) **Clustering of 20 samples of five healthy subjects. Samples were clustered using UPGMA with weighted UniFrac as a distance measure. The figure was generated with the interactive Tree of Life tool (iTOL) [70]. Participating volunteers are numbered HV1 to HV5 followed by the sampled body location. Colored bars represent the relative abundance of bacterial genera as determined by barcoded pyrosequencing (details in Materials and methods). **(b) **Phylogenetic diversity rarefaction curves for communities sampled from the listed skin locations show differences between armpit, forehead, inner elbow and upper buttock skin.

The upper buttock is a standard location for invasive procedures to study skin injury in human subjects [11]. As this body site was not included in previous microbiome studies [18], we first analyzed its microbiome composition for comparison with other known locations. We obtained samples from five healthy volunteers in order to analyze the microbial composition of the upper buttock skin, the forehead (sebaceous environment) and from two moist environments of the body (armpit and inner elbow). In total, 116,291 bacterial 16S rRNA sequences were analyzed (Additional file 1). Hierarchical clustering using weighted UniFrac as distance measure (details in the Materials and methods) separates samples from different body environments into three groups (indicated by the numbers 1 to 3 in Figure 1a), with all armpit samples in group 1, 4 out of 5 forehead samples in group 2, and 4 out 5 samples from the upper buttock in group 3 (Figure 1a). Notably, the samples of the inner elbow did not cluster together and appeared divided over the three groups. The phylogenetic composition is distinct for upper buttock skin compared to the other three body sites with the exception of individual HV2 (Figure 1a; Additional file 2). We found that the upper buttock had the largest bacterial diversity of the analyzed sites: rarefaction curves show that the phylogenetic diversity observed in different body sites is highest in upper buttock skin for this limited number of samples (Figure 1b). In the moist region of the armpit we identified a high proportion of reads assigned to the Firmicutes phylum (72.4%) and a lower fraction of reads assigned to the Actinobacteria phylum (27.2%), whereas the sebaceous environment of the forehead was dominated by the Actinobacteria phylum (90.7%). At other locations of the skin the relative abundances of the phyla Firmicutes and Actinobacteria were differently distributed (respectively 30.7% versus 65.7% on the inner elbow, and 41.3% versus 46.4% on the upper buttock). Furthermore, the higher microbial diversity on upper buttock skin was reflected by the detection of other, relatively low abundant phyla, such as Proteobacteria (10.3%), Bacteroidetes (1.3%), Cyanobacteria (0.1%) and Acidobacteria (< 0.1%).

### Influence of the individual, gender and stratum corneum depth on microbiota composition of the upper buttock skin

**Figure 2:**
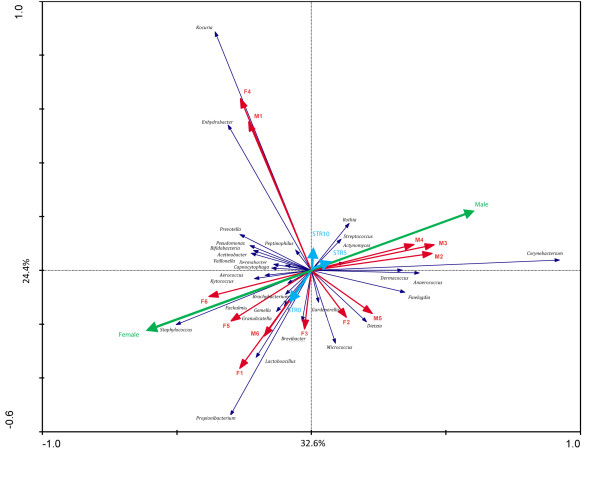
**Redundancy analysis of the microbiota composition of the lower back of 12 adults for determining the most important variables (17 in total) explaining the variation in microbiota composition at the genus level**. Genera that represented at least 85.1% of the first two principal components used as explanatory axis in the plots are shown as vectors. The first component and second component explain 32.6% and 24.4% of the variance, respectively. This figure was generated with Canoco version 4.5. The different variables are represented by arrows, where length reflects significance. Colors indicate sample groupings: red arrows represent all individuals, green arrows represent the males and females, and the blue arrows correspond to the stripping depth (STR0 is non-damaged healthy superficial skin, and STR5 and STR10 represent 5 and 10 times tape-stripped skin, respectively). The microbial genera are shown in black text color.

To investigate the composition of microbial communities in different stratum corneum layers we sampled the skin of 12 healthy volunteers at different depths following repeated tape stripping as a method of mechanical removal of stratum corneum layers (for detailed description see Materials and methods and Additional file 3). Samples for microbiome analysis were subsequently taken by swabbing normal skin and the deeper layers of the stratum corneum as exposed by tape stripping. In total, 495,709 bacterial 16S rRNA sequences were analyzed, resulting in an average of 5,901 (range 2,430 to 12,035) reads per sample (Additional file 4). The effect of volunteer origin, gender and stripping depth on microbiota composition were analyzed by redundant analysis, revealing volunteer-specific signatures (Figure 2, red arrows pointing to distinct directions) and strong effect of gender (Figure 2, green arrows) on microbiota composition. Redundant analysis also indicated important differences in microbial composition between the superficial and the deeper layer of the stratum corneum (blue arrows).

**Figure 3:**
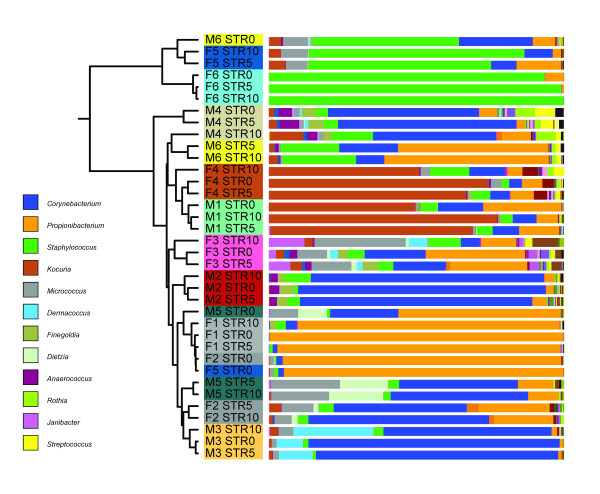
**Clustering and microbial community composition of different volunteers and epidermal layers**. Samples were clustered using UPGMA with weighted UniFrac as a distance measure. The figure was generated with iTOL [70]. Sample names with the same color come from the same volunteer (M = male 1 to 6, F = female 1 to 6), followed by the stripping depth (STR0, STR5, STR10). Colored bars represent the relative abundance (the number of reads assigned to a genus divided by the total number of reads assigned up to the phylum level) of bacterial genera as determined by barcoded pyrosequencing.

**Figure 4:**
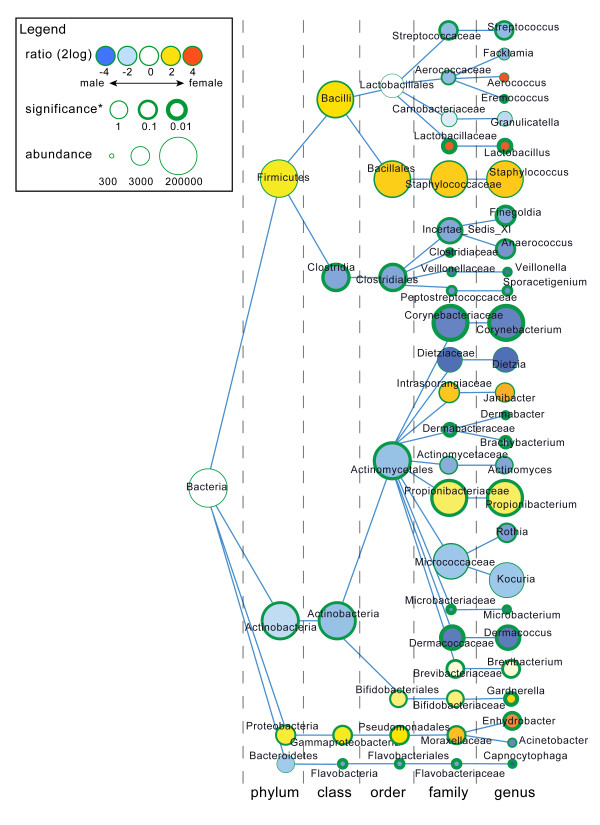
**Difference in microbial community composition between males and females**. Nodes represent taxa, edges link the different taxonomic levels. The fold increase is calculated as the 2log of the ratio of the relative in males and females (0 = no difference between genders, 1 = twice as abundant in female, and so on). The significance is expressed as the *P*-value of a Mann-Whitney U test of the male and female samples. Note that the relation between node-size and total abundance is non-linear.

**Figure 5:**
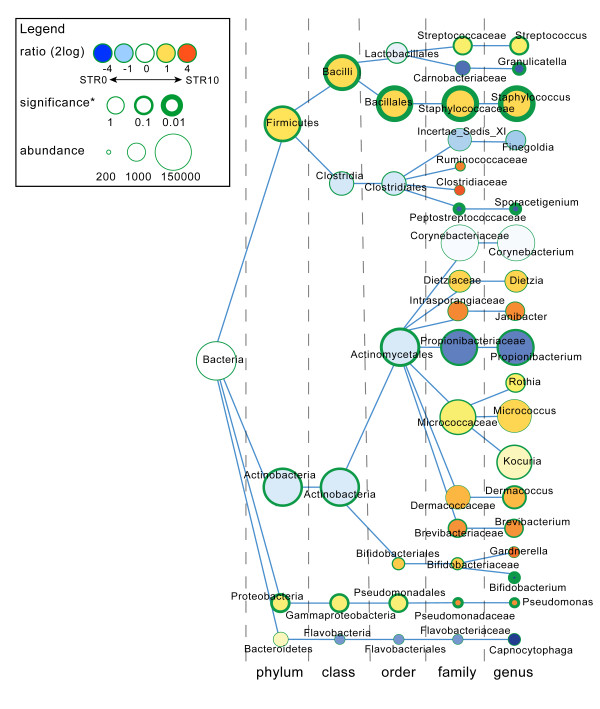
**Differences in microbial community composition between STR0 and STR10 samples**. Nodes represent taxa, edges link the different taxonomic levels. The fold increase is calculated as the 2log of the ratio of the relative in males and females (0 = no difference between STR0 and STR10, 1 = twice as abundant in STR10, and so on). The significance is expressed as the *P*-value of a Mann-Whitney U test of the male and female samples. Note that the relation between node size and total abundance is non-linear.

Hierarchical clustering of all samples showed that the individual volunteer is the strongest denominator of microbiota composition (Figure 3). UniFrac analysis showed no distinct clustering caused by either tape stripping or gender variables. However, even though the signal is small compared to the differences between volunteers, a small, but potential biologically relevant, signal could easily be obscured by the noise introduced by the individual nature of the samples. In-depth analysis of gender differences revealed bacterial taxa that were significantly different in relative abundance, and broadly supported gender differences in microbiota composition of the upper buttock skin (Figure 4). Males have relatively high proportions of *Streptococcus, Eremococcus, Finegoldia, Anaerococcus, Veillonella, Sporacetigenium, Corynebacterium, Dermabacter, Brachybacterium, Microbacterium, Dermacoccus *and *Capnocytophaga *when compared to females. Furthermore, females have relatively high proportions of *Lactobacillus, Propionibacterium, Gardnerella *and *Enhydrobacter*. The significance of these differences was determined using a Mann-Whitney U rank test (Figure 4). There is also a tendency for higher ratios of *Staphylococcus, Janibacter *and *Brevibacterium *in females. Most interestingly, we identified enrichment of *Lactobacillus *and *Gardnerella *to be specific for females and *Corynebacterium *in males. In addition, one of the *Lactobacillus *operational taxonomic units (OTUs) associated with females (*P*-value 0.034) could be assigned to the vaginal inhabitant *Lactobacillus iners*, as a microbial BLAST search showed the sequence to be 100% identical over the full 337 nucleotides to this species, validating the biological relevance of our statistical approach. Overall, males showed a higher bacterial diversity of the microbiome compared to females (Table 1) for all four diversity metrics tested (Shannon, Chao1, whole tree phylogenetic diversity, and observed OTUs), reflected by the presence of more small bacterial groups in males (Figure 4). Because we have observed significant differences in microbial composition between the superficial and deeper layers of the stratum corneum (Figure 2), we also used taxonomy-based classification provided by the Ribosomal Database Project combined with statistical mining to identify significant differences in microbiota composition (Figure 5). In the deeper layers of the stratum corneum (STR5 and STR10) we found a relative increase in the proportion of the following genera: *Streptococcus, Staphylococcus, Rothia, Kocuria, Dermacoccus, Brevibacterium, Bifidobacterium *and *Pseudomonas*. Furthermore, a relative decrease of *Granulicatella, Propionibacterium *and *Sporacetigenium *was found in these deeper layers. Translated to the phylum level, an increase in the relative abundance of Firmicutes (represented by *Staphylococcus*) in the deeper layers of the stratum corneum and a decrease of Actinobacteria (*Propionibacterium*) was observed (Figure 5).

**Table 1:** Differences in microbial diversity of superficial skin microbiota of men and women

	Alpha diversity^a^			
	
Volunteer	Phylogenetic diversity	Observed OTUs	Shannon	Chao1
F1	3.1	81.8	1.4	170.7
F2	4.4	98.4	1.5	230.0
F3	15.0	264.8	5.5	561.7
F4	10.4	178.6	3.2	350.7
F5	3.8	75.2	1.5	174.8
F6	2.1	48.0	0.9	90.0
Mean female	6.5	124.5	2.3	263.0
M1	10.9	167.2	3.5	340.5
M2	9.6	172.6	3.9	360.3
M3	4.6	94.2	2.4	183.9
M4	14.6	246.6	5.4	519.0
M5	7.5	151.8	3.3	337.4
M6	11.7	210.2	4.0	416.2
Mean male	9.8	173.8	3.8	359.6
Significance^b^	0.075	0.131	0.039	0.1

^a^Calculated after rarification to 2,194 reads per sample, average of 5 rarification trials. ^b^*P*-value of rank test (Mann-Whitney U) of the samples from male volunteers versus the samples from female volunteers.

### Recolonization patterns of the microbiome after skin barrier disruption

**Figure 6:**
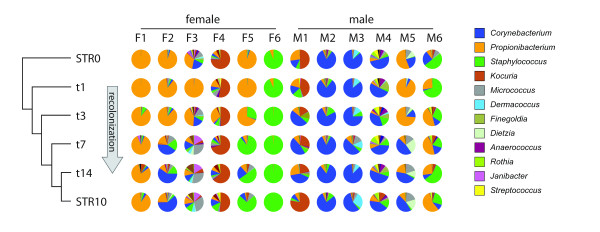
**Recolonization after stripping**. The tree-like structure in the left side of the figure is the consensus tree. It was generated using consense (Phylip_REF) of the per-volunteer UPGMA UniFrac trees of the samples. The pie charts show the microbial community composition of the individual samples. Composition is displayed as relative abundance, that is, the number of reads assigned to a genus divided by the total number of reads assigned up to the genus level.

We investigated the temporal changes in the microbiome composition of the skin surface following mechanical removal of the stratum corneum by tape stripping. Again, the host seems to be the most important denominator of microbiota composition as most of the samples were grouped by individual using hierarchical UniFrac clustering (Additional file 5). Initially, we observed that the microbiota profile of injured skin is considerably disturbed up to day 14 when compared to the microbiome of the superficial skin layer before stripping (Figure 6). It has to be noted that tape-stripped skin at day 14 in general has fully recovered from the initial injury as assessed by clinical (not shown) and microscopic criteria (Additional file 6). Even if the samples from different volunteers differ widely in their constituent taxa, the behavior and dynamics in time can be similar. The consensus tree shows that after tape stripping, the microbiota composition (visualized by the pie charts in Figure 6) on day 1 reflects the superficial layer, which then diverges within 14 days in the direction of the STR10 state. No difference between partial or complete removal of the stratum corneum was found with respect to microbiome changes in the process of recolonization. This is illustrated by the pie charts, which respectively correspond to 15 times tape-stripped skin (F1 to F3 and M1 to M3) and skin with a completely removed stratum corneum (F4 to F6 and M4 to M6). Gender differences also appeared in the recolonization process during the first days after tape stripping, as we observed a relative increase of *Propionibacterium*, which was a more dominant bacterial group in women, at the cost of *Staphylococcus, Corynebacterium *and *Micrococcus*. The microbiome shifts in response to skin injury were less dramatic in males than in females (Additional file 7).

### Large interindividual variation of epidermal antimicrobial protein expression following injury of human skin

**Figure 7:**
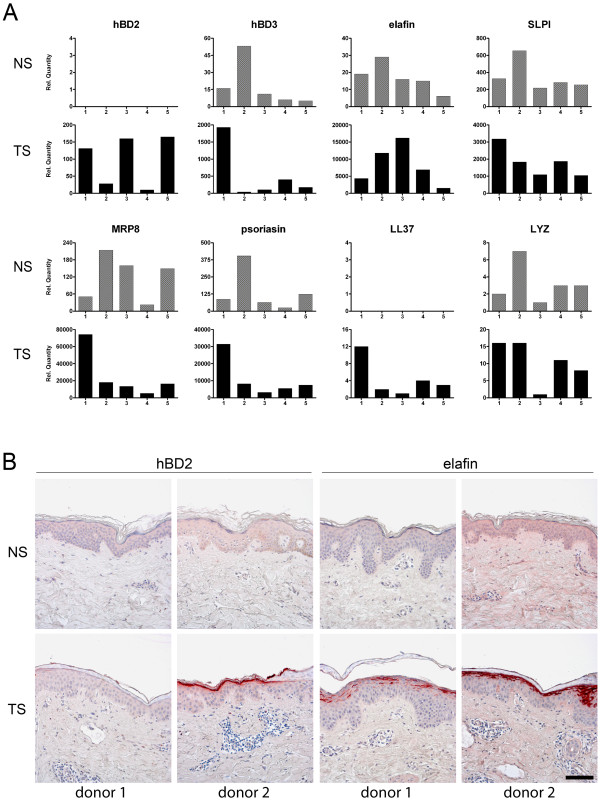
**Variation in the antimicrobial protein host response upon superficial skin injury**. **(a) **Variation in epidermal mRNA expression levels of eight genes that encode antimicrobial proteins, in normal healthy skin (NS) and upon tape stripping (TS) after 24 hours (n = 5). **(b) **hBD-2 and elafin protein expression levels before and after tape stripping in several individuals. Scale bar = 100 μm.

As reported above and described in earlier studies, the microbiota composition varies considerably between individuals [15,17,18]. This applies both to diversity and the dominance of certain taxonomic groups, for example, as shown for females F1 (*Propionibacterium *dominance) and F6 (*Staphylococcus *dominance) in Figure 6. The factors that determine the skin microbiome composition are not known, but host factors involved in innate immunity of the skin are likely to play a role in shaping the microbiome under steady state conditions and following disturbance of homeostasis (for example, by skin injury). Previous work from our lab has shown that expression of antimicrobial proteins is strongly induced following skin barrier disruption [11,40,41]. In this part of the study we compared the responses of five healthy controls with respect to induction of eight different antimicrobial proteins. Note that these individuals were distinct from the volunteers in the tape stripping study from whom no biopsy material was available. Gene expression profiles at baseline and 24 hours after tape stripping of the upper buttock skin were analyzed by quantitative PCR (qPCR). In normal healthy skin antimicrobial proteins are expressed at moderate levels (SLPI, MRP8, psoriasin), low levels (hBD-3, elafin, lysozyme) or undetectable levels (hBD-2, LL37) (Figure 7a). An indication for the basal antimicrobial protein expression levels are the mean Ct values for *SLPI *(Ct = 26), MRP8 (*S100A8*, Ct = 27), psoriasin (*S100A7*, Ct = 28), hBD-3 (*DEFB103*, Ct = 30), elafin (*PI3*, Ct = 28), lysozyme (*LYZ*, Ct = 33), hBD-2 (*DEFB4*, Ct = 37) and LL37(*CAMP*, Ct = 37). Upon tape stripping, all genes showed upregulation, with the highest fold-increases for elafin, MRP8 and psoriasin. Although the eight genes showed induction in all individuals studied, we observed a hitherto unrecognized large variation between individuals. For example, in individual 1 there was abundant induction of all antimicrobial protein genes except for elafin, whereas individual 3 showed low responses for hBD-3, SLPI, MRP8, psoriasin, LL37, and lysozyme compared to the other individuals while the expression levels of hBD-2 and elafin are the highest in this person. This phenomenon was also confirmed at the protein level (Figure 7b), where hBD-2 and elafin protein expression levels at 48 hours after tape stripping varied between individuals.

## Discussion

Bacterial colonization of human skin starts during birth and continues throughout the first years of life. The microbial communities then stabilize and contribute to the establishment of cutaneous homeostasis and modulation of innate immune responses [42]. The temporary or permanent presence of these microbes on our skin depends on the topographical regions of the body with their own specific conditions (for example, pH, moisture and sebum content), host-specific factors (for example, age and sex), and environmental factors specific for the individual (for example, occupation, lifestyle, geographical location, antibiotics use, the use of cosmetics and soaps) [12]. Until now, samples for microbiome analyses have been taken under static conditions from the outer skin surface [15-18,42]. Intriguingly, the results of the present study suggest that the microbiome of the deeper stratum corneum layers plays an important role in the microbial recolonization process of the skin after injury. In addition, microbiome dynamics of human epidermis following skin barrier disruption showed high interpersonal variation, and important gender differences. Our data on host innate immune response suggested variation in antimicrobial protein expression following superficial injury.

We first compared the microbial community compositions of the upper buttock skin, forehead, armpit and inner elbow. The upper buttock skin contains a high bacterial diversity when compared to forehead, armpit and inner elbow. The most prominent genera in the upper buttock skin were *Staphylococcus, Propionibacterium, Micrococcus, Corynebacterium, Enhydrobacter *(which belongs to phylum Proteobacteria), and *Streptococcus*. Compared to the study of Grice *et al. *[18], who sampled the back between the scapulae and the (lower) buttock, our data show that the microbial composition of the upper buttock is intermediate between these two, although it more resembles the back skin. *Propionibacteria *predominated the sebaceous environment of the back between the scapula, which we also found in significant proportions on the upper buttock skin. In contrast to this, upper buttock skin contained smaller proportions of Proteobacteria (Additional file 8).

Previous studies have already suggested gender differences of normal skin [16,43]. In our current study, microbiome dynamics of human epidermis following skin barrier disruption also revealed pronounced differences between males and females. Several physiological and anatomical gender differences that influence skin properties, such as hormone production, sweat rate, sebum production, surface pH, skin thickness and hair growth [44], could account for the microbial differences observed between genders. In a large pyrosequencing-based study on the bacterial composition of the hand surface, it was shown that men and women harbor distinct bacterial communities. Women showed a significantly greater bacterial diversity than men, even when controlling for hand hygiene, and these differences between genders become more apparent with time following hand washing [16]. Given the observation that men generally have a more acidic skin surface than women, it was speculated that differences in skin pH may be influential as microbial diversity is often lower in more acidic environments. These gender differences may also impact on behavioral characteristics (for example, use of cosmetics) that influence the bacterial communities found on the hands. This was also proposed in another molecular analysis of bacterial community composition (based on 16S rRNA gene clone sequencing), which revealed significantly higher bacterial diversity between forehead samples from men compared to women [43]. However, when samples from women using make-up were excluded, these gender differences were no longer observed, suggesting that the use of make-up strongly interferes with the microbiota composition [43]. Remarkably, in our current study, men showed a higher diversity of microbiota composition on the upper buttock skin compared to females. These experiments revealed that bacteria are not uniformly distributed in the stratum corneum but are clearly different between the superficial and deeper layers of human skin (Figure 2). Furthermore, the extent of microbiota disturbances observed during the first days after stripping was greater in females (Additional file 7). One could speculate that the gender differences in microbial community composition as reported by others [16,43] may be due to sampling of the superficial layer of the skin, which could be considered more as a reflection of host/gender-specific environmental factors (sebum, sweat, washing behavior, cosmetic use). We found that the microbiome of injured skin is considerably disturbed during the entire 14-day period of the study when compared with the microbiota composition obtained from the surface of uninjured skin. With this knowledge, we would recommend to follow microbiome dynamics in injured skin for a longer period in similar future studies. However, when the microbial community composition of the deeper stratum corneum layer (STR10) is regarded as the host indigenous microbiome, the composition at day 14 after injury is very similar to this situation (Figure 6). Our data imply that differences in microbial composition between the sexes exist, at least in the upper buttock skin, that are not attributable to specific environmental circumstances that influence the local microbiome. Surprisingly, common inhabitants of the reproductive organs like *Lactobacillus *and *Gardnerella *in females and *Corynebacterium *in males were identified. Bacteria that normally reside on the labia minora and the glans penis [15,27] are now identified on the upper buttock skin (and also in the deeper layers of the stratum corneum), indicating that microbes known to colonize specific anatomical locations may also spread and occupy other body parts.

Chronic nonhealing wounds affecting diabetic, elderly and immobilized individuals are often infected or colonized, and cause significant morbidity and economic burden [12,13,45]. It is thought that these chronic wounds are not initially caused by bacteria, although they might negatively affect healing of infected wounds. The exact role of the human skin microbiome in the pathogenesis of chronic wounds is unclear. Several molecular-based studies reported a wide range of microorganisms in chronic wounds with different underlying conditions (for example, diabetes, venous disease, decubitus, high blood pressure, non-healing postoperative wounds), but no specific organisms that colonize wounds of the same etiology were identified [46,47]. It was suggested that fastidious anaerobes have a critical function in the pathogenesis of chronic wounds [45]. Our current study, in which we characterized the dynamics of microbial communities in healing superficial wounds of 12 healthy individuals, revealed that *Propionibacterium*, a typically anaerobic bacterium, was found to be a dominant genus in the early recolonization phase. Recently, a longitudinal shift in diabetic wound microbiota was found to correlate with aberrant innate immunity gene expression in mice [38]. It was also reported that the normal skin microbiota supports and modulates the innate immune host response to prevent colonization of potentially pathogenic microorganisms [22,23]. Therefore, it is plausible that investigation of the interrelationship between human cutaneous microbiome and the host immune system will lead to a better understanding of the dynamics of wound healing [48]. Recently, it was shown that resident microbiota are necessary for optimal skin immune fitness [49]. It was found that cutaneous commensal bacteria exert their affect by augmenting IL-1 signaling and consequently effector T-cell function, which is relevant knowing that IL-1 is implicated in the etiology and pathology of psoriasis and other cutaneous disorders [50].

Examination of antimicrobial protein gene expression profiles in tape-stripped skin revealed an unrecognized large interindividual variation (Figure 7). Speculatively, the individual-specific levels of expression of the innate antimicrobial genes could account for the host-specific microbial communities that are exposed to the superficial wounds in the early recolonization phase. As the data on antimicrobial protein expression were obtained from a different set of individuals to the individuals of the microbiome study, we could not analyze the correlations between host response repertoire and microbiota. This is clearly an interesting question, although addressing this issue may run into practical and ethical problems in view of the potentially large number of individuals that need to be subjected to tape stripping and biopsy-taking and the strict inclusion criteria. As discussed earlier, the composition of these microbial communities could be influenced by environmental circumstances and/or result from (epi-)genetic variations that exist between each individual that might create a selective environment that favors or eliminates specific microorganisms. Recent studies have identified genetic risk factors for psoriasis and atopic dermatitis that affect skin barrier function [51,52]. In addition, we have previously reported that the expression of antimicrobial proteins is also dependent on genetically programmed differences [53]. An aberrant skin barrier function might result in exposure of epidermal cells to environmental microbial components, which could evoke an inflammatory response, shaped by the genetic background.

## Conclusions

**Figure 8:**
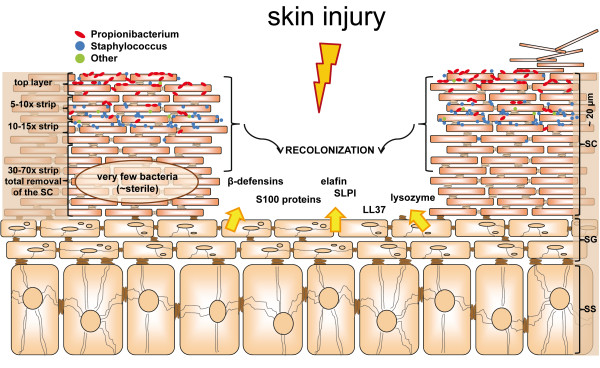
**Model for skin injury, microbial recolonization, and host response**. Details in Discussion and Conclusion sections. Recolonization is done by bacteria from the deeper layers of the adjacent skin (black arrows). During this process the microbial communities of the host and invading bacteria from the environment trigger the skin to express antimicrobial proteins and inflammatory molecules (yellow arrows). SC = stratum corneum; SG = stratum granulosum; SS = stratum spinosum.

The main focus of our research was to identify the dynamics of the skin microbiota and recolonization behavior after skin damage. In addition to these findings, our study provides a first overview of the specific bacterial players involved, which should be considered as leads for larger and more detailed analysis. Based on our findings we here present a working model that there is a short-lived recolonization of the damaged skin with microbial constituents (frequently *Propionibacterium*) from the surrounding superficial skin layer (bacteria from adjacent skin) and that this transient microbiome is replaced by the microbiome that inhabits the deeper layers of the stratum corneum (Figure 8). During the recolonization process the microbial communities of the host and invading bacteria from the environment trigger the skin to express antimicrobial proteins and inflammatory molecules. These host-specific innate immune responses may help the skin in closing the wound, resulting in a restored barrier function in which epidermal keratinocytes are in homeostasis with the local microbiome.

In a recent review by Virgin and Todd [54], it was hypothesized that microbial communities influence our resistance and susceptibility to multifactorial inflammatory diseases like type 1 diabetes, ulcerative colitis and Crohn's disease. It was postulated that disease genetics may be combinatorial with different host-gene-microbial interactions, contributing to the pathogenesis of disease in subsets of patients. It is known that there are different pathways to the same diagnosis in complex diseases, which is supported by the observation that subsets of patients respond differently to mechanistically distinct interventions. These considerations also apply to common skin diseases such as atopic dermatitis and psoriasis. Both diseases show clinical variability with respect to disease manifestation and therapeutic responses, which could be linked to differential involvement of skin microbiota (*Staphylococcus aureus *and group A streptococci, respectively). Understanding of host-gene-microbial interactions could possibly lead to the identification of mechanistically important interactions that enable more accurate interpretation of genetics, pathogenesis, and therapeutic success [54]. The finding that null alleles of the epidermis-expressed filaggrin (*FLG*) gene are a major risk factor for atopic dermatitis [52], and our observation that copy number variation of epidermis-expressed genes such as β-defensins and LCE3B/C predispose to psoriasis [51,55], indicate that epidermal biology and stratum corneum homeostasis play an important role in these common inflammatory diseases. Our present study demonstrates quantitative and qualitative differences between the microbiomes of the various stratum corneum layers. Most bacteria reside in the upper layers as bacterial DNA was undetectable in the deeper layers (> 15 times stripping; Additional file 9). Whereas intact stratum corneum appears to be an effective barrier to colonization of the deeper stratum corneum layers, this may be quite different in skin conditions with disturbed barrier function (for example, atopic dermatitis and psoriasis). The question is if genetically determined variation of stratum corneum properties leads to shifts in bacterial communities at a high hierarchical level (for example, phylum) or rather favors the colonization by particular species. Microbiota may be differently distributed or even qualitatively different, as suggested by a recent study on psoriasis [35]. Speculatively, this may lead to abnormal exposure of the epidermal keratinocytes or Langerhans cells to live bacteria or bacterial components. Continuous exposure to pathogen-associated molecular patterns (PAMPs) may lead to uncontrolled stimulation of pattern recognition receptors (PRR) some of which were shown to be abnormally expressed in lesional psoriasis skin [56]. Stimulation of the innate and adaptive immune system by PAMPs or by specific antigens could be a driving force for the chronic inflammatory process, but such a scenario clearly requires experimental confirmation.

The study we present here provides essential leads and suggestions for the design of more in-depth studies. Such investigations could include targeted identification of microbial taxa and host factors that regulate the microbiome composition in normal skin, but also during wound healing or in skin diseases. Such studies will contribute to understand the relationship between host and microorganisms, and may lead to novel strategies in prevention and development of new therapies for treatment. Selective modulation of skin microbiota composition by pre- and/or probiotica [57] could be interesting future strategies to achieve beneficial effects in patients.

## Materials and methods

### Ethics statement

All volunteers in this study were selected according to the inclusion/exclusion criteria as approved by a protocol from the National Institutes of Health (NIH) Human Microbiome Project (protocol number HMP-07-001, 30 March 2009; protocol available at the NIH Human Microbiome Project website) [58]. The exact inclusion/exclusion criteria and study procedures that we presented to the volunteers can be found in Additional files 10 and 11. Medical ethical committee (Commissie Mensgebonden Onderzoek Arnhem-Nijmegen) approval and individual written informed consent were obtained in advance of sample collection. The study was conducted according to the Declaration of Helsinki principles.

### Study participants and sample collection

Five healthy volunteers (one male and four females, Caucasian, aged 26 to 31 year) were included in the topographical study. Subjects were instructed not to wash or use body lotion for 24 hours prior to sampling and to avoid swimming in a chlorinated pool, using a hot tub, sauna/steam bath or tanning bed for 48 hours prior to sampling (Additional file 11). All samples were taken in April 2010. Samples were collected from body sites with different microenvironments: two moist regions (armpit, inner elbow), a sebaceous region (forehead) and a presumed dry region (upper buttock). Samples were obtained by swabbing 4 cm^2 ^skin areas using Sterile Catch-All™ Sample Collection Swabs (Epicentre Biotechnologies, Madison, WI) soaked in sterile SCF-1 solution (50 mM Tris buffer (pH8), 1 mM EDTA, and 0.5% Tween-20). The swabbing technique was executed as follows: stretch the skin with one hand while the other hand holds the swab so that the shaft is parallel to the skin surface. Subsequently, the swab is rubbed back and forth approximately 50 times applying firm pressure. Immediately after swabbing, each swab is swirled in a 2 ml collection tube containing 300 μl MicroBead Solution (MO BIO Laboratories, Carlsbad, CA). The swab sponge should be pressed against the tube wall multiple times for 20 seconds to ensure transfer of bacteria from swab to solution. Samples were stored at -20°C until further processing. To minimize sample cross-contamination, a fresh pair of sterile gloves was worn by the person sampling each individual.

To study microbiome dynamics of human epidermis following skin barrier disruption we used the well-described 'tape stripping' method [10]. This method involves the repeated application of adhesive tape to the skin surface, thereby removing stratum corneum layers. This method creates a superficial wound showing slight skin irritation (erythema) and loss of barrier function (transepidermal water loss). An example of the morphology of tape-stripped skin and subsequent regeneration of human epidermis is depicted in hematoxylin and eosin stained sections in Additional file 6. For this study (all samples were obtained in February 2011), we recruited 12 healthy Caucasian volunteers, 6 male and 6 female, aged 21 to 56 years, according to the Human Microbiome Project criteria as described above. Four of these volunteers participated also in the topographical study (Additional file 12). For tape stripping experiments we selected the upper buttock skin (just under the waistband) for two reasons. First, this area of the body contains a relatively high richness of observed OTUs (Results section; Figure 1; Additional files 1 and 2). In addition, the skin at this body site is a convenient area for tape stripping experiments or biopsy taking, which may sometimes result in abnormal wound healing with poor cosmetic results.

To study the human skin microbiome in different epidermal layers, two areas on the right upper buttock measuring 2 cm^2 ^(1 × 2 cm) each were tape-stripped 5 (STR5) and 10 (STR10) times, respectively, by application and removal of adhesive tape (n = 12). Using the swabbing technique described above, the two barrier-disrupted skin areas were sampled, as well as 2 cm^2 ^healthy non-barrier disrupted (STR0) right upper buttock skin (see Additional file 3 for experimental set-up).

To study recolonization of the human skin microbiome after skin barrier disruption, four areas on the left upper buttock measuring 2 cm^2 ^each were tape-stripped 15 times (n = 6, three males and three females) or as many times as required to obtain a glistening surface, which indicates complete removal of the stratum corneum. The latter procedure (usually 30 to 70 times tape stripping) exposes the upper living epidermal cell layer and was performed in six individuals, three males and three females. These tape-stripped areas were sampled over time for up to two weeks (see Additional file 3 for experimental set-up). This end point was chosen for the reason that, in general, injured skin (by tape stripping) recovers completely within this time period based on clinical and microscopic criteria. In a previous pilot experiment we found that 15 times tape stripping of healthy upper buttock skin minimizes bacterial counts below detectable levels with qPCR (Additional file 9). Analysis of 15 times tape-stripped skin up to complete removal of the stratum corneum shows that this lower compartment is practically devoid of detectable bacterial DNA. The reason for including skin samples with totally removed stratum corneum (30 to 70 times tape stripping) is that this results in a moist wound surface, which may have other recolonization characteristics than the mildly tape-stripped (15 times) wounds.

Finally, we analyzed archival material of five healthy volunteers from which we have obtained biopsies from the left upper buttock (males and females, Caucasian race, aged 24 to 66 years) to study the host response towards superficial injury. These individuals were previously tape-stripped on two areas on the upper buttock measuring 4 cm^2 ^each until the surface became slightly shiney. After 24 and 48 hours, 3-mm biopsies were taken from the tape-stripped area and from healthy skin for both RNA isolation and histology, respectively, to examine antimicrobial protein gene expression profiles of the host.

### DNA extraction

DNA was extracted from the swabs using the MO BIO Ultraclean Microbial DNA Isolation Kit with modifications. As described above, each swab was swirled in a 2 ml collection tube containing 300 μl MicroBead Solution. Subsequently, 50 μl MD1 Solution was added to the bacterial cells and the samples were heated for 10 minutes at 70°C. This bacterial solution was transferred to a MicroBead tube and horizontally vortexed for 10 minutes at maximum speed using the MO BIO Vortex Adapter tube holder. The remaining steps were performed as described in the manual as provided by the manufacturer. DNA samples (30 μl in MD5 solution) were stored at -20°C until further processing.

### Choice of universal primers

The following universal primers were applied for amplification of the V3-V6 region of the 16S rRNA gene: forward primer, 5'-*CCATCTCATCCCTGCGTGTCTCCGACTCAGNNNNNN***ACTCCTACGGGAGGCAGCAG**-3' (the italicized sequence is 454 Life Sciences primer A, and the bold sequence is the broadly conserved bacterial primer 338F; *NNNNNN *designates the sample-specific six-base barcode used to tag each PCR product); reverse primer 5'-*CCTATCCCCTGTGTGCCTTGGCAGTCTCAG***CRRCACGAGCTGACGAC**-3' (the italicized sequence is 454 Life Sciences primer B, and the bold sequence is the broadly conserved bacterial primer 1061R).

### PCR amplification and sample preparation

Data from a pilot experiment revealed that samples derived from 2 cm^2 ^of skin on the upper buttock contain only small numbers of microorganisms (just a slight band around 800 bp of length on agarose gel after 16S rRNA gene PCR amplification). Therefore, we introduced a pre-amplification step with the same primers as described above excluding the barcodes and flag sequences. In this pilot experiment we established that this additional PCR step did not affect the results compared to a single amplification step with bar-coded primers; in other words, we observed no distortion or skewing of the taxa distribution. As a negative control we used a mock swab, which was only exposed to ambient air, and extracted the genomic microbial DNA from this sample. However, even after two rounds of PCR no visible band on agarose gel was observed. The first amplification PCR consists of 5 μl microbial genomic DNA, 16 μl master mix (1 μl KOD Hot Start DNA Polymerase (1 U/μl; Novagen, Madison, WI, USA), 5 μl KOD-buffer (10 ×), 3 μl MgSO4 (25 mM), 5 μl dNTP mix (2 mM each), 1 μl (10 μM) of each forward and reverse primer), and 29 μl sterile water (total volume 50 μl). PCR conditions were: 94°C for 2 minutes followed by 30 cycles of 94°C for 20 s, 55°C for 10 s, and 70°C for 15 s, ending with a last step of 72°C for 10 minutes to ensure complete amplification of the target region. The approximately 750 bp PCR amplicon was subsequently purified using the MSB Spin PCRapace kit (Invitek, Westburg, The Netherlands) and the concentration was checked with a Nanodrop 2000 spectrophotometer (Thermo Scientific, Wilmington, DE). All PCR amplicons were diluted to a concentration of 10 ng/μl, and 3 μl of this purified amplicon (total 30 ng) was used as input for a re-amplification PCR using the bar-coded primers as described above. PCR conditions were: 94°C for 2 minutes followed by 35 cycles of 94°C for 20 s, 55°C for 10 s, and 70°C for 15 s, ending with a last step of 70°C for 10 minutes. Again, the approximately 750 bp PCR product was purified (MSB Spin PCRapace kit) and the concentration was measured. A composite sample for pyrosequencing was prepared by pooling 100 ng of these purified PCR products of each sample. The pooled sample was elecrophorized on a 1% agarose gel and the approximately 800 bp band was excised and extracted from the agarose gel with the MinElute Gel Extraction kit (Qiagen, Venlo, The Netherlands). The concentration of the gel-extracted amplicon was determined and 50 μl (concentration 14.5 ng/μl purified PCR product) was submitted for pyrosequencing of the V3-V4 region of the 16S rRNA gene on the 454 Life Sciences GS-FLX platform using Titanium sequencing chemistry at DNAvision, Charleroi, Belgium.

### 16S rRNA gene sequence analysis

Pyrosequencing data were analyzed with a workflow based on QIIME v1.2 [59], using settings as recommended in the QIIME 1.2 tutorial, with the following exceptions: reads were filtered for chimeric sequences using Chimera Slayer [60]; the number of QC-passed sequences per sample are presented in Additional file 4 and OTU clustering was performed with settings as recommended in the QIIME newsletter of 17 December 2010 [61] using an identity threshold of 97%. Diversity metrics were calculated as implemented in QIIME 1.2. Hierarchical clustering of samples was performed using UPGMA with weighted UniFrac as a distance measure as implemented in QIIME 1.2. The Ribosomal Database Project classifier version 2.2 was performed for taxonomic classification [62]. Visualization of differences in relative abundance of taxa between different sample groups (Figures 4 and 5) was done in Cytoscape [63]. Taxa (that is, nodes) were included in the visualization if they met the following criteria: all samples together have at least 10 reads assigned to the taxon and the sample groups have a fold-difference of at least 0.1 for the taxon, or the taxon has a child (that is, more specific taxonomic classification) meeting the first criterion. The significance of the difference in relative abundance of specific taxa between sample groups was calculated using the Mann-Whitney U test as implemented in SciPy [64]. We provide additional information on the diversity metrics and statistics in Additional file 13. Additional data handling was done using in-house developed Python and Perl scripts.

### Isolation of epidermal sheaths, RNA extraction and real-time qPCR

Isolation of epidermal sheets for mRNA extraction was performed as previously described [65]. RNA was extracted using the RNeasy Mini Kit (Qiagen, Hilden, Germany). A DNase I treatment was performed according to the manufacturer's protocol (Invitrogen, Carlsbad, CA, USA). Reverse transcriptase reactions and qPCR were performed as described previously [39]. The amount of mRNA for a given gene in each sample was normalized to the amount of mRNA of the human ribosomal phosphoprotein P0 (RPLP0) reference gene in the same sample. Primers for qPCR (Biolegio, Nijmegen, the Netherlands) were only accepted if their efficiency was 100 ± 10%. Corrections were made for primer efficiency. Primer sequences and efficiency are shown in Additional file 14. Relative mRNA expression levels were calculated with the delta-delta Cycle threshold (ΔΔCt) method [66]. In case of very low to absent mRNA levels (Ct value > 37), the relative quantity was set to 0. All mRNA expression levels in purified epidermal sheets are related to the lysozyme expression of the normal healthy skin of volunteer 3, which was set to 1.

### Immunohistochemistry

Skin biopsies were immediately fixed in a 10% formalin solution (Baker Mallinckrodt, Deventer, The Netherlands) for 4 hours and subsequently embedded in paraffin. Sections were stained with hematoxylin and eosin or analyzed by immunohistochemistry as follows: sections were blocked for 15 minutes with 20% normal rabbit serum (for hBD-2) or normal goat serum (elafin) in phosphate-buffered saline and subsequently incubated with anti-hBD-2 (1:100; Abcam, Cambridge, UK), and anti-elafin (1:500) for 1 hour at room temperature. Next, sections were incubated for 30 minutes with a secondary antibody (biotinylated rabbit anti-goat or biotinylated goat anti-rabbit in phosphate-buffered saline containing 1% bovine serum albumin, Vector laboratories, Burlingame, CA, USA). After 30 minutes incubation with Avidin-Biotin complex (Vector Laboratories, Burlingame, CA), sections were treated with 3-amino-9-ethyl carbazole (Calbiochem, San Diego, CA, USA) for 10 minutes.

### Data availability

Sequence data from this study have been submitted to MG-RAST [67] as project number 2329 [68]. The barcodes for linking reads in the pooled samples to individual samples are provided in Additional file 4.

## Abbreviations

IL: interleukin; iTOL: interactive tree of life tool; OTU: operational taxonomic unit; PAMP: pathogen-associated molecular pattern; qPCR: quantitative PCR.

## Competing interests

The authors declare that they have no competing interests.

## Authors' contributions

Conceived and designed the experiments: PLZ, PMK, JS. Performed the experiments: PLZ, EHB, HDK, IIS. Analyzed the data: PLZ, JB, DMS, SH, MK, JS, HMT. Wrote the paper: PLZ, JB, JS, HMT. All authors have read and approved the manuscript.

## Supplementary Material

Additional file 1 **Table showing microbiome analysis of 4 different body locations (N = 5)**. Click here for file

Additional file 2 **Microbial community composition of upper buttock skin and forehead**. Relative abundances of the most abundant bacterial taxa on (A) upper buttock, and (B) forehead. The figure illustrates the average composition of the sample for different levels of bacterial taxonomy, from the domain level (left) to the genes level (right). Figure generated using software described in Sundquist *et al *[69], also providing or more detailed description of the visualization. Click here for file

Additional file 3 **Experimental set-up tape-stripping upper buttock skin**. On the right site the experiment is depicted that was used to study the human skin microbiome in different epidermal layers. Two areas on the right upper buttock measuring 2 cm^2 ^(1 × 2 cm) each, were tape-stripped 5 (STR5) and 10 (STR10) times respectively by application and removal of adhesive tape (n = 12, F1-6 and M1-6). Subsequently, barrier disrupted skin areas were sampled, as well as 2 cm^2 ^healthy non-barrier disrupted right upper buttock skin (STR0). To study recolonization of the human skin microbiome after skin barrier disruption, four areas on the left upper buttock measuring 2 cm^2 ^each, were tape-stripped 15 times (n = 6, F1-3 and M1-3) or as many times as required to obtain a glistening surface which indicates complete removal of the stratum corneum (F4-6 and M4-6). Click here for file

Additional file 4 **Table with read and OTU counts**. Click here for file

Additional file 5 **Clustering and microbial community composition of different volunteers and recolonization in time**. Samples were clustered using UPGMA with weighted UniFrac as a distance measure. The figure was generated with iTOL [70]. Composition is displayed as relative abundance, i.e. the number of reads assigned to a genus divided by the total number of reads assigned up to the genus level. Sample names with the same color come from the same volunteer (M = male1 to 6, F = female1 to 6), followed by the time of recolonization (t = 1, 3, 7 or 14 days). STR0 is normal, healthy skin (not tape-stripped). Colored bars represent the relative abundance of bacterial genera as determined by barcoded pyrosequencing. Click here for file

Additional file 6 **Morphology of tape-stripped skin and subsequent regeneration of human epidermis**. (A) H&E staining of normal healthy skin. (B) The stratum corneum which is present in normal skin has been stripped off completely (biopsy taken 2 hours after tape-stripping). (C) A picture taken 4 hours after tape-stripping showing more hypertrophic basal cells, several pyknotic nuclei in cells of the stratum spinosum, and a layer of parakeratotic cells that begins to form on the surface. (D) At the stage of 24 hours after tape-stripping the basal cells are really hypertrophic and these columnar basal cells make up about one third of the thickness of the epidermis. Hyperparakeratosis is observed on top of the epidermis. (E) Pronounced acanthosis and hyperparakeratosis is seen after 48 hours. At this stage the tape-stripping skin model resembles most lesional psoriatic skin. (F) Finally at 96 hours, the hyperparakeratosis is disappeared and a fresh anuclear stratum corneum is formed on the emerging stratum granulosum. Scale bar = 100 μm. Click here for file

Additional file 7 **Relative abundance of all detected genera in females (A) and males (B) of the deeper skin layer (STR10) compared to the recolonizing skin in time (DAY 1, 3, 7 and 14)**. Click here for file

Additional file 8 **Topographical distribution of bacteria on skin sites on the back**. The upper buttock skin contains a high bacterial diversity (data from the present study) and its microbial composition is intermediate between the microbiome of the back between the scapulae and the lower buttock as published by Grice *et al *[18]. Moist sites are labeled in green, sebaceous sites are labeled in blue, and dry surfaces in red. The upper buttock is labeled in purple. Click here for file

Additional file 9 **Yield bacterial genomic DNA after tape-stripping**. Swabs were taken from upper buttock skin and from skin that was tape-stripped 1, 10, 15, and 30 times on this part of the body. Genomic DNA was extracted using the Mobio Ultraclean Microbial DNA Isolation Kit and concentrations were determined by real-time qPCR using broad range universal primers targeting the 16S rRNA gene [71] and calculated from a standard dilution series of *Staphylococcus epidermidis*. Click here for file

Additional file 10 **Exclusion criteria**. Description of the exact inclusion/exclusion criteria. Click here for file

Additional file 11 **Study procedures**. Description of the study procedures presented to the volunteers. Click here for file

Additional file 12 **Participation of volunteers**. Specification of which volunteers participated in the different studies. Click here for file

Additional file 13 **Supplementary methods**. The text outlines the different statistical tools used in our analysis. Click here for file

Additional file 14 **Table with qPCR primer sequences and efficiency**. Click here for file
