# Supplementary material for: Microbiome dynamics of human epidermis following skin barrier disruption
Source: Genome Biol. 2012 Nov 15;13(11):R101. doi: 10.1186/gb-2012-13-11-r101 (PMC3580493; doi:10.1186/gb-2012-13-11-r101)
Supplement: Additional file 1 — Table showing microbiome analysis of 4 different body locations (N = 5). [file gb-2012-13-11-r101-S1.PDF]

Microbiome analysis of 4 different body locations (N=5)

UPPER BUTTOCK	READS	PERCENTAGE
**Total reads**	24662	100
**Assigned at genus level**	23347	94.7
**Assigned at phylum level**	24569	99.6

INNER ELBOW	READS	PERCENTAGE
**Total reads**	30793	100
**Assigned at genus level**	29387	95.4
**Assigned at phylum level**	30669	99.6

FOREHEAD	READS	PERCENTAGE
**Total reads**	30124	100
**Assigned at genus level**	29193	96.9
**Assigned at phylum level**	29909	99.3

ARMPIT	READS	PERCENTAGE
**Total reads**	30712	100
**Assigned at genus level**	30216	98.4
**Assigned at phylum level**	30610	99.7
