# Supplementary figures and images for: Microbiome dynamics of human epidermis following skin barrier disruption
Source: Genome Biol. 2012 Nov 15;13(11):R101. doi: 10.1186/gb-2012-13-11-r101 (PMC3580493; doi:10.1186/gb-2012-13-11-r101)

**Tape-strip**  
swab on:  
day 1  
day 3  
day 7  
day 14

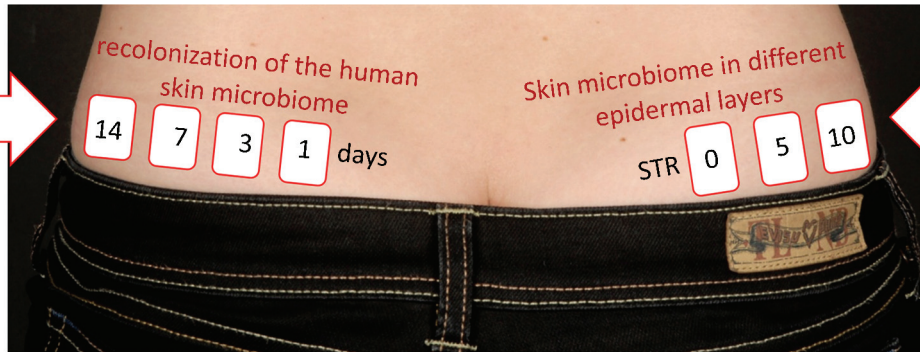

**STR-0x**  
**STR-5x**  
**STR-10x**  
swab on:  
day 0

Supplement: Additional file 3 — Experimental set-up tape-stripping upper buttock skin. On the right site the experiment is depicted that was used to study the human skin microbiome in different epidermal layers. Two areas on the right upper buttock measuring 2 cm2 (1 × 2 cm) each, were tape-stripped 5 (STR5) and 10 (STR10) times respectively by application and removal of adhesive tape (n = 12, F1-6 and M1-6). Subsequently, barrier disrupted skin areas were sampled, as well as 2 cm2 healthy non-barrier disrupted right upper buttock skin (STR0). To study recolonization of the human skin microbiome after skin barrier disruption, four areas on the left upper buttock measuring 2 cm2 each, were tape-stripped 15 times (n = 6, F1-3 and M1-3) or as many times as required to obtain a glistening surface which indicates complete removal of the stratum corneum (F4-6 and M4-6). [file gb-2012-13-11-r101-S3.PDF]

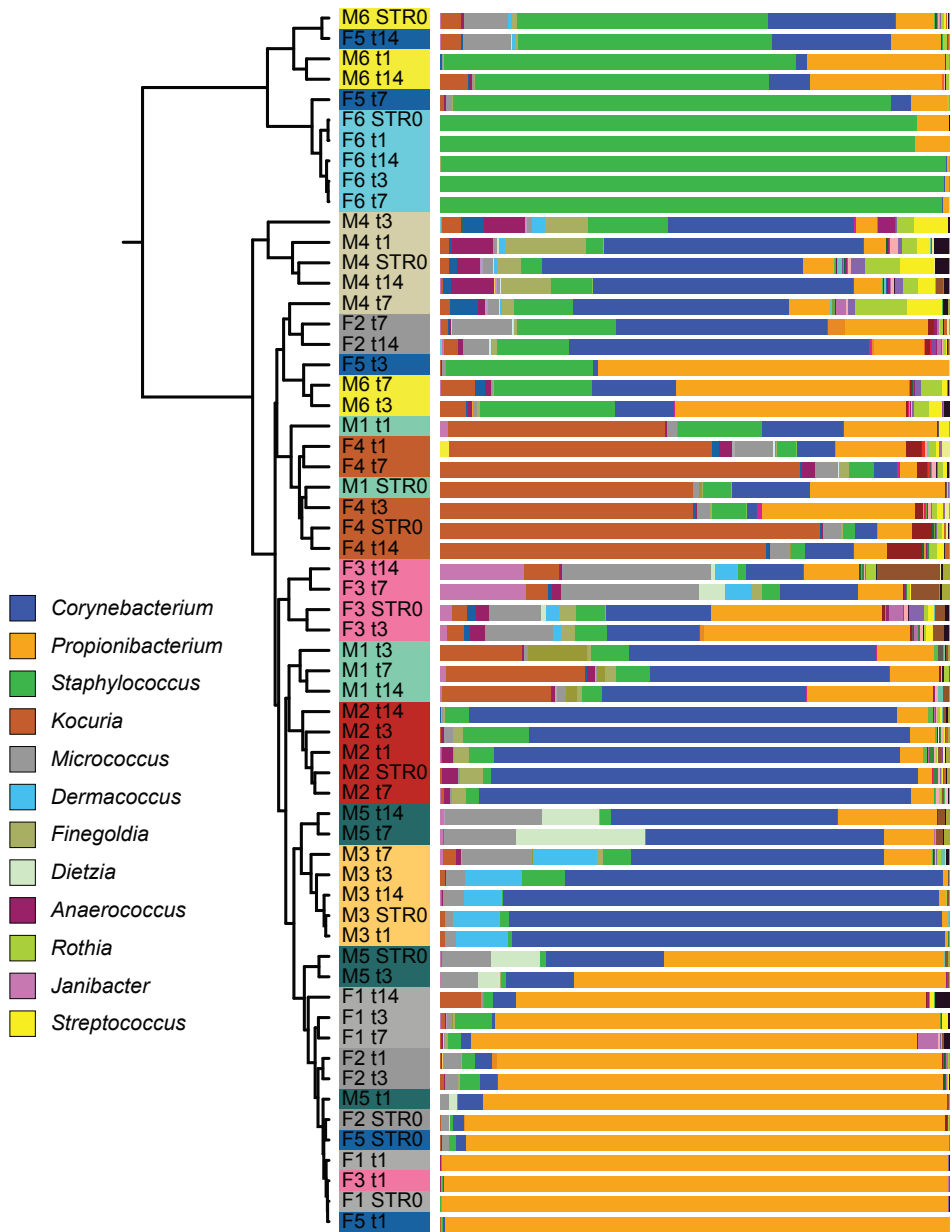

Supplement: Additional file 5 — Clustering and microbial community composition of different volunteers and recolonization in time. Samples were clustered using UPGMA with weighted UniFrac as a distance measure. The figure was generated with iTOL [70]. Composition is displayed as relative abundance, i.e. the number of reads assigned to a genus divided by the total number of reads assigned up to the genus level. Sample names with the same color come from the same volunteer (M = male1 to 6, F = female1 to 6), followed by the time of recolonization (t = 1, 3, 7 or 14 days). STR0 is normal, healthy skin (not tape-stripped). Colored bars represent the relative abundance of bacterial genera as determined by barcoded pyrosequencing. [file gb-2012-13-11-r101-S5.PDF]

A

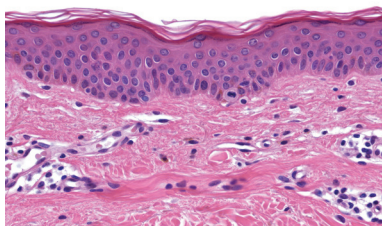

B

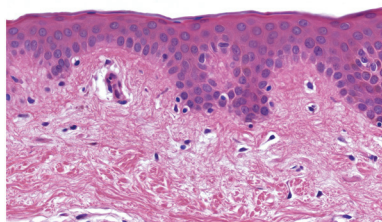

C

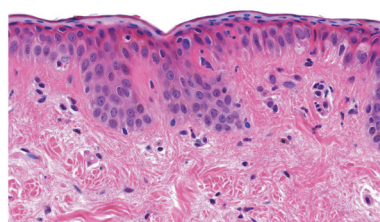

D

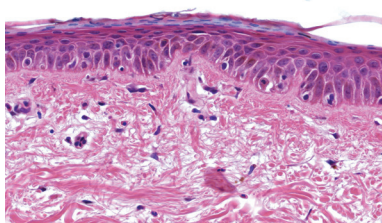

E

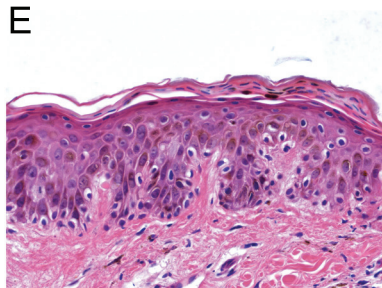

F

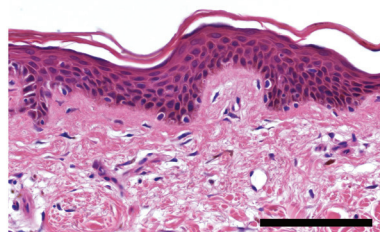

Supplement: Additional file 6 — Morphology of tape-stripped skin and subsequent regeneration of human epidermis. (A) H&E staining of normal healthy skin. (B) The stratum corneum which is present in normal skin has been stripped off completely (biopsy taken 2 hours after tape-stripping). (C) A picture taken 4 hours after tape-stripping showing more hypertrophic basal cells, several pyknotic nuclei in cells of the stratum spinosum, and a layer of parakeratotic cells that begins to form on the surface. (D) At the stage of 24 hours after tape-stripping the basal cells are really hypertrophic and these columnar basal cells make up about one third of the thickness of the epidermis. Hyperparakeratosis is observed on top of the epidermis. (E) Pronounced acanthosis and hyperparakeratosis is seen after 48 hours. At this stage the tape-stripping skin model resembles most lesional psoriatic skin. (F) Finally at 96 hours, the hyperparakeratosis is disappeared and a fresh anuclear stratum corneum is formed on the emerging stratum granulosum. Scale bar = 100 μm. [file gb-2012-13-11-r101-S6.PDF]

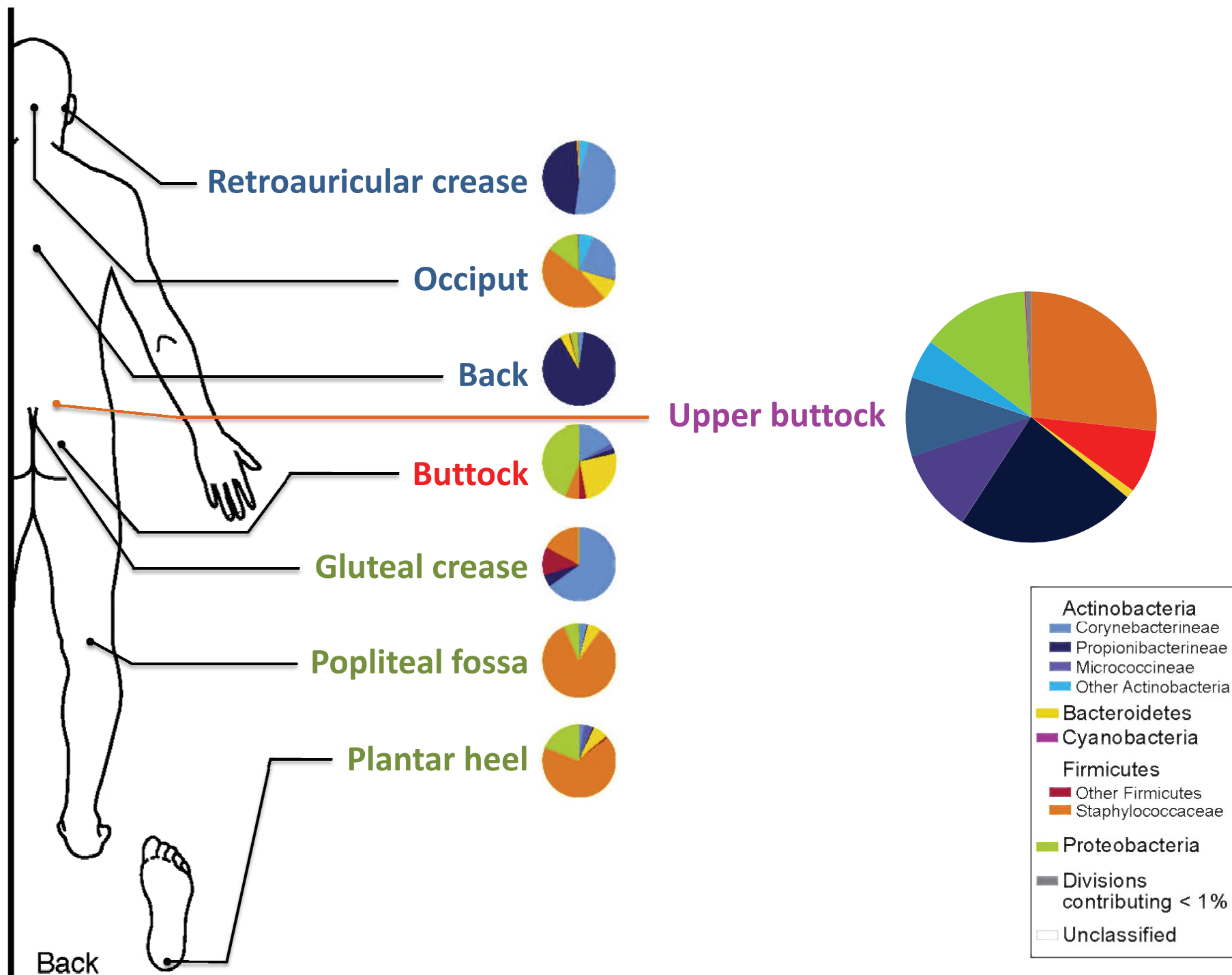

Supplement: Additional file 8 — Topographical distribution of bacteria on skin sites on the back. The upper buttock skin contains a high bacterial diversity (data from the present study) and its microbial composition is intermediate between the microbiome of the back between the scapulae and the lower buttock as published by Grice et al [18]. Moist sites are labeled in green, sebaceous sites are labeled in blue, and dry surfaces in red. The upper buttock is labeled in purple. [file gb-2012-13-11-r101-S8.PDF]

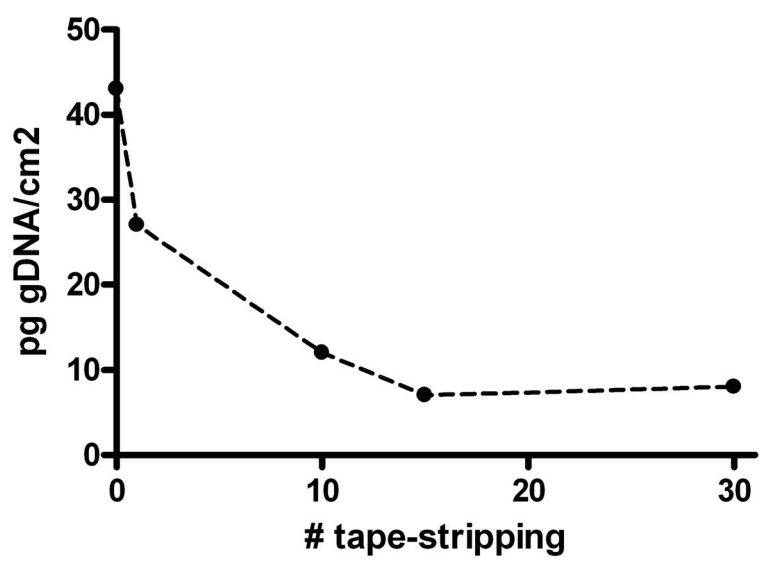

Supplement: Additional file 9 — Yield bacterial genomic DNA after tape-stripping. Swabs were taken from upper buttock skin and from skin that was tape-stripped 1, 10, 15, and 30 times on this part of the body. Genomic DNA was extracted using the Mobio Ultraclean Microbial DNA Isolation Kit and concentrations were determined by real-time qPCR using broad range universal primers targeting the 16S rRNA gene [71] and calculated from a standard dilution series of Staphylococcus epidermidis. [file gb-2012-13-11-r101-S9.PDF]
