# Supplementary material for: Microbiome dynamics of human epidermis following skin barrier disruption
Source: Genome Biol. 2012 Nov 15;13(11):R101. doi: 10.1186/gb-2012-13-11-r101 (PMC3580493; doi:10.1186/gb-2012-13-11-r101)
Supplement: Additional file 4 — Table with read and OTU counts. [file gb-2012-13-11-r101-S4.PDF]

Read and OTU counts

VOLUNTEER	SAMPLE	READS[1]	OTUs[2]	BARCODE[3]
F1	STR0	8099	175	aagcgt
F1	STR5	7021	176	aaggcc
F1	STR10	5284	210	aaggtt
F1	STR15-1	7685	128	aagtgc
F1	STR15-3	6298	206	aagtat
F1	STR15-7	5495	209	aagtcg
F1	STR15-14	3616	160	aaggag
F2	STR0	6885	177	aataag
F2	STR5	5442	319	aatatt
F2	STR10	3420	252	aatcat
F2	STR15-1	5615	215	aatgac
F2	STR15-3	4413	191	aatcca
F2	STR15-7	2816	293	aatcgc
F2	STR15-14	4849	300	aataga
F3	STR0	4685	393	aatggt
F3	STR5	4101	378	acaacc
F3	STR10	5205	356	acaatg
F3	STR15-1	8283	131	acagag
F3	STR15-3	4432	392	acacga
F3	STR15-7	4870	329	acactt
F3	STR15-14	7456	456	aatgta
F4	STR0	6809	315	acagct
F4	STR5	4167	218	acatac
F4	STR10	4666	227	acatca
F4	STR40-1	4497	350	accata
F4	STR40-3	5361	335	accaat
F4	STR40-7	5365	419	accagg
F4	STR40-14	3972	267	acagtc
F5	STR0	6542	148	accgcg
F5	STR5	5828	223	acctcc
F5	STR10	5431	183	acctgt
F5	STR40-1	7655	118	acgaga
F5	STR40-3	6292	182	accttg
F5	STR40-7	7636	183	acgact
F5	STR40-14	3419	300	accgga
F6	STR0	8036	98	acgcat
F6	STR5	9114	56	acgcgc
F6	STR10	11473	56	acggac
F6	STR40-1	9708	86	acgtgg
F6	STR40-3	8619	61	acggca
F6	STR40-7	12035	82	acgtaa
F6	STR40-14	11052	66	acgccg
M1	STR0	4706	250	acgttc
M1	STR5	6587	281	actacg
M1	STR10	5888	238	actagt
M1	STR15-1	5299	321	actctc
M1	STR15-3	4741	293	actcaa
M1	STR15-7	4888	350	actcgg
M1	STR15-14	4089	297	actaac
M2	STR0	5312	277	actgat

M2	STR5	6408	316	actgtg
M2	STR10	4129	228	acttag
M2	STR15-1	5876	328	agaact
M2	STR15-3	5471	286	acttct
M2	STR15-7	5639	284	acttga
M2	STR15-14	5440	317	actgcc
M3	STR0	7091	179	agacca
M3	STR5	6752	192	agactc
M3	STR10	6084	260	agagat
M3	STR15-1	7804	218	agatcg
M3	STR15-3	6448	216	agaggc
M3	STR15-7	5974	420	agagtg
M3	STR15-14	6511	194	agacgg
M4	STR0	4055	351	agatta
M4	STR5	4714	308	agcaga
M4	STR10	4473	302	agcatt
M4	STR40-1	4007	322	agcgcc
M4	STR40-3	2430	217	agccaa
M4	STR40-7	4385	369	agccgt
M4	STR40-14	4020	364	agcaac
M5	STR0	7159	301	agcgta
M5	STR5	6012	307	agctca
M5	STR10	6496	286	aggaat
M5	STR40-1	3433	118	aggcac
M5	STR40-3	6801	253	aggagc
M5	STR40-7	7618	304	aggatg
M5	STR40-14	6741	358	agctat
M6	STR0	6203	366	aggcta
M6	STR5	5573	325	aggtct
M6	STR10	4571	233	aggtga
M6	STR40-1	5411	154	agtcga
M6	STR40-3	6408	337	agtaca
M6	STR40-7	4941	296	agtcag
M6	STR40-14	5474	305	aggtag

[1]Number of reads after quality control and removal of chimeric sequences.
[2]Number of OTUs at an identity threshold of 97%
[3]The six-nucleotide tag used to identify individual samples in the pooled sequencing reads
