# Supplementary material for: Microbiome dynamics of human epidermis following skin barrier disruption
Source: Genome Biol. 2012 Nov 15;13(11):R101. doi: 10.1186/gb-2012-13-11-r101 (PMC3580493; doi:10.1186/gb-2012-13-11-r101)
Supplement: Additional file 7 — Relative abundance of all detected genera in females (A) and males (B) of the deeper skin layer (STR10) compared to the recolonizing skin in time (DAY 1, 3, 7 and 14). [file gb-2012-13-11-r101-S7.PDF]

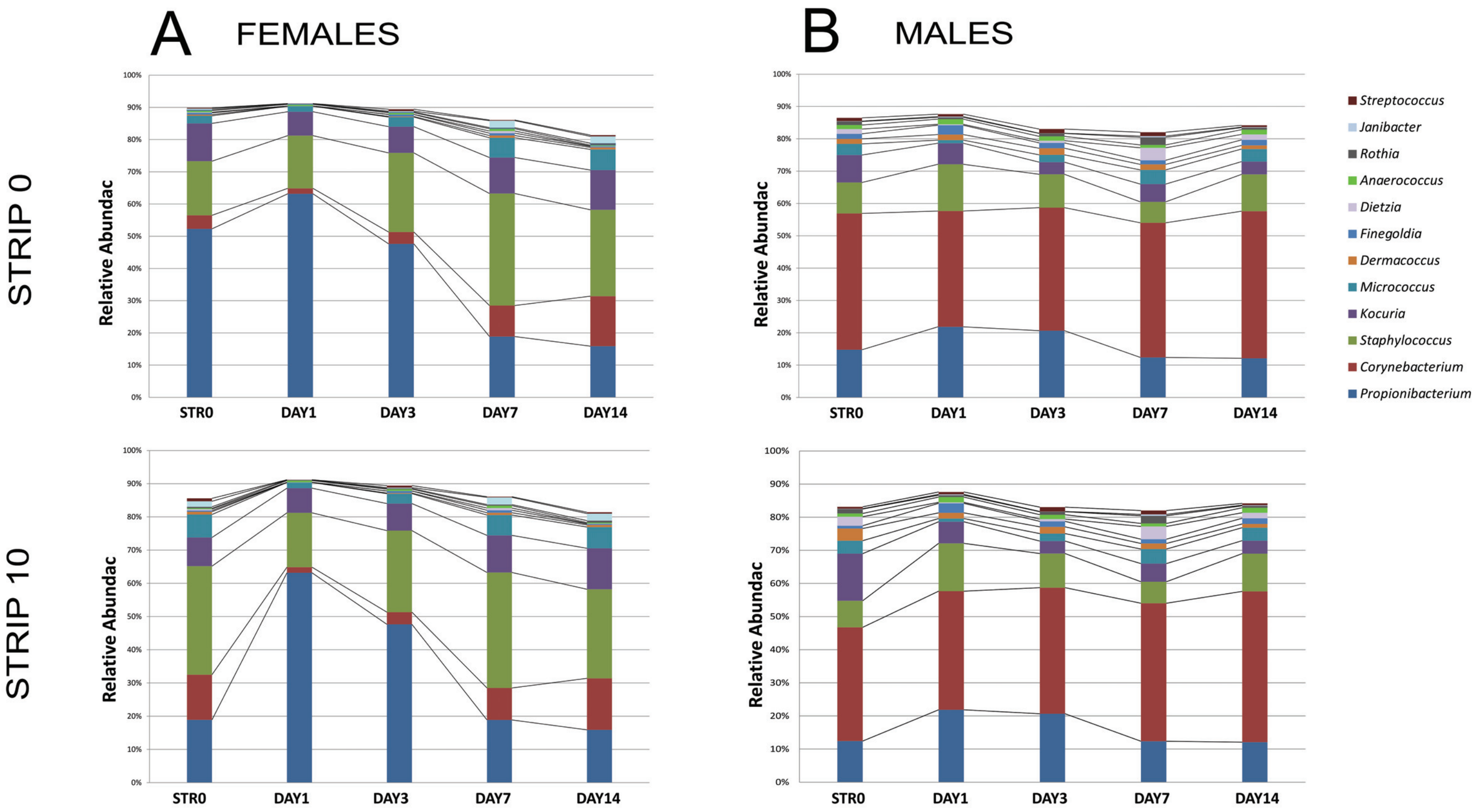
A
FEMALES
B
MALES
STRIP 0
STRIP 10
Relative Abundac
100%
90%
80%
70%
60%
50%
40%
30%
20%
10%
0%
STR0
DAY1
DAY3
DAY7
DAY14
Streptococcus
Janibacter
Rothia
Anaerococcus
Dietzia
Finegoldia
Dermacoccus
Micrococcus
Kocuria
Staphylococcus
Corynebacterium
Propionibacterium
