# Supplementary material for: Microbiome dynamics of human epidermis following skin barrier disruption
Source: Genome Biol. 2012 Nov 15;13(11):R101. doi: 10.1186/gb-2012-13-11-r101 (PMC3580493; doi:10.1186/gb-2012-13-11-r101)
Supplement: Additional file 10 — Exclusion criteria. Description of the exact inclusion/exclusion criteria. [file gb-2012-13-11-r101-S10.PDF]

Exclusion criteria

Any subject who meets any of the following criteria will be excluded from participation in this study:

- Body Mass Index greater than or equal to 35 or less than or equal to 18.
- Use of any of the following drugs within the last 6 months:
    - systemic antibiotics (intravenous, intramuscular, or oral);
    - oral, intravenous, intramuscular, nasal or inhaled corticosteroids;
    - cytokines;
    - methotrexate or immunosuppressive cytotoxic agents;
    - large doses of commercial probiotics consumed (greater than or equal to 108 cfu or organisms per day) - includes tablets, capsules, lozenges, chewing gum or powders in which probiotic is a primary component. Ordinary dietary components such as fermented beverages/milks, yogurts, foods do not apply.
- Use of topical antibiotics, antifungal or topical steroids within the previous 7 days.
- Acute disease at the time of enrolment (defer sampling until subject recovers). Acute disease is defined as the presence of a moderate or severe illness with or without fever.
- Chronic, clinically significant (unresolved, requiring on-going medical management or medication) pulmonary, cardiovascular, gastrointestinal, hepatic or renal functional abnormality, as determined by medical history or physical examination.
- History of cancer except for squamous or basal cell carcinomas of the skin that have been medically managed by local excision.
- Unstable dietary history as defined by major changes in diet during the previous month, where the subject has eliminated or significantly increased a major food group in the diet.
- Recent history of chronic alcohol consumption defined as more than five 1.5-ounce servings of 80 proof distilled spirits, five 12-ounce servings of beer or five 5-ounce servings of wine per day.
- Confirmed positive test for HIV, HBV or HCV.
- Any confirmed or suspected condition/state of immunosuppression or immunodeficiency (primary or acquired) including HIV infection.
- Major surgery of the GI tract, with the exception of cholecystectomy and appendectomy, in the past five years. Any major bowel resection at any time.
- History of active uncontrolled gastrointestinal disorders or diseases including:
    - inflammatory bowel disease (IBD) including ulcerative colitis (mildmoderate-severe), Crohn's disease (mild-moderate-severe), or indeterminate colitis;
    - irritable bowel syndrome (IBS) (moderate-severe);

- persistent, infectious gastroenteritis, colitis or gastritis, persistent or chronic diarrhea of unknown etiology, *Clostridium difficile* infection (recurrent) or *Helicobacter pylori* infection (untreated);
- chronic constipation.

• Female who is pregnant or lactating.

• Treatment for or suspicion of ever having had toxic shock syndrome.

• History of psoriasis or recurrent eczema.

• History of recurrent rashes within the past 6 months.

• At the time of the screening visit or on the specimen collection day:

▪ acne at sites other than on the face, chest, back or shoulders;

▪ multiple blisters, pustules, boils, abscesses, erosions or ulcers on the scalp, face, neck, arms, forearms or hands;

▪ a single blister, pustule, boil, abscess, erosion, ulcer, scab, cut, crack or pink/hyperpigmented patch or plaque at or within 4 cm of the sampling sites; sampling may be deferred until the lesion resolves either without treatment or with local treatment only;

▪ more than one pink/red scaly patch/plaque anywhere on the body (suggestive of psoriasis or eczema);

▪ uniformly thickened, cracking, “dry” skin on bilateral palms and/or soles;

▪ scalp dandruff that does not clear up with over-the-counter dandruff shampoos used daily for 2 weeks;

▪ disseminated rash (at multiple body sites or extending throughout a broad body area).

• Chronic dry mouth.

• Periodontitis / gingivitis.

• Evidence of untreated cavitated carious lesions or oral abscesses.

• Evidence of precancerous or cancerous oral lesions.

• Evidence of oral candidiasis.

• More than 8 missing teeth. The missing teeth must be due to 3rd molar extractions and/or teeth extracted for orthodontic purposes, teeth extracted as a result of trauma, or teeth that are congenitally missing.
