# Supplementary material for: Microbiome dynamics of human epidermis following skin barrier disruption
Source: Genome Biol. 2012 Nov 15;13(11):R101. doi: 10.1186/gb-2012-13-11-r101 (PMC3580493; doi:10.1186/gb-2012-13-11-r101)
Supplement: Additional file 11 — Study procedures. Description of the study procedures presented to the volunteers. [file gb-2012-13-11-r101-S11.PDF]

Study procedures

• Subjects have to refrain from swimming in a chlorinated pool or using a hot tub for 48 hours prior to sampling visit.
• Avoid the use of sauna/steam baths for 48 hours prior to sampling visit.
• Avoid the use of tanning bed for 48 hours prior to sampling visit.
• Bathe/shower procedure:
  ▪ First stripping/swab on Monday 14 February (2011), then swab on Tuesday 15 February (day 1), on Thursday 17 February (day 3), on Monday 21 February (day 7) and on Monday 28 February (day 14).
  ▪ Do NOT shower on these days (you can wash your hair but avoid leaking soap to your back skin).
  ▪ On days you shower do NOT rub up your back skin (avoid soap as much as possible).
  ▪ Do NOT scrub your back skin with a towel, please dab a little to dry your back.
  ▪ Avoid the use of body lotion on the lower back during the experiment.
