# Supplementary material for: Microbiome dynamics of human epidermis following skin barrier disruption
Source: Genome Biol. 2012 Nov 15;13(11):R101. doi: 10.1186/gb-2012-13-11-r101 (PMC3580493; doi:10.1186/gb-2012-13-11-r101)
Supplement: Additional file 12 — Participation of volunteers. Specification of which volunteers participated in the different studies. [file gb-2012-13-11-r101-S12.PDF]

Participation of volunteers

Person number	Sex (M/F)	Age (years)	Topographical study	Tape stripping study	Antimicrobial gene expression study
1	F	30	**HV1**	x	x
2	F	27	**HV2**	**F6**	x
3	F	26	**HV3**	**F5**	x
4	M	31	**HV4**	**M1**	x
5	F	26	**HV5**	**F2**	x
6	M	31	x	**M2**	x
7	M	21	x	**M3**	x
8	M	56	x	**M4**	x
9	M	55	x	**M5**	x
10	M	40	x	**M6**	x
11	F	27	x	**F1**	x
12	F	24	x	**F3**	x
13	F	36	x	**F4**	x
14	F	56	x	x	**1**
15	M	64	x	x	**2**
16	M	59	x	x	**3**
17	F	66	x	x	**4**
18	F	24	x	x	**5**
