# Supplementary material for: Microbiome dynamics of human epidermis following skin barrier disruption
Source: Genome Biol. 2012 Nov 15;13(11):R101. doi: 10.1186/gb-2012-13-11-r101 (PMC3580493; doi:10.1186/gb-2012-13-11-r101)
Supplement: Additional file 13 — Supplementary methods. The text outlines the different statistical tools used in our analysis. [file gb-2012-13-11-r101-S13.PDF]

Supplementary methods

In the research described in this paper we analyze different types of data and mine this data for a variety of biological signals. This necessitates the use of different metrics and statistical tools. In this section, we outline the different diversity metrics and methods used to determine the significance of differences community compositions we observe.

Diversity

Alpha diversity

Alpha diversity reflects the diversity within a sample based on the abundance of various taxa within a community. Qiime provides tools for the calculation of a wide variety of metrics. One of these metrics is "Phylogenetic Distance", a metric reflecting the fraction of the microbial tree covered by a sample. This metric considers phylogeny in determining diversity, in contrast to other metrics, which only consider OTU counts, ignoring the information that some OTUs are more closely related than others. Figure 1B use the Phylogenetic Distance measure to express diversity. In our analysis of the difference in microbial diversity of male and female skin, we provided diversity expressed in Phylogenetics, as well as 3 other commonly used metrics (Table 1), as these other metrics are more familiar to many.

Beta diversity

Beta diversity involves the comparison of microbial communities based on their composition. Beta-diversity metrics describe the difference between microbial communities. Throughout the paper we use the metric UniFrac, which measures the phylogenetic distance between sets of taxa in a phylogenetic tree as the fraction of the branch length of the tree that leads to descendants from either one environment or the other, but not both [1].

Significance

The significance of putative differences in relative abundance of specific tax between sample groups (e.g. male – female) is expressed as the p-value of a Mann-Whitney U rank test. This test takes all samples (i.e. the combined male and female set) and ranks them based on the relative abundance of the taxon of interest. The null hypothesis of this test: there is no relation between the sample group (male or female) and the relative abundance of the taxon of interest, therefore the different groups should be well-mixed in the ranked list of samples. If this is not the case (in the case of male – female and the taxon *Lactobacillus*: the female samples are on the high end of the ranking), the null hypothesis is rejected, indicating a putative link (note that this link does not have to be causal).

References

1. Lozupone C, Knight R: **UniFrac: a new phylogenetic method for comparing microbial communities.** *Appl Environ Microbiol* 2005, **71:**8228-8235.
