# Supplementary material for: Microbiome dynamics of human epidermis following skin barrier disruption
Source: Genome Biol. 2012 Nov 15;13(11):R101. doi: 10.1186/gb-2012-13-11-r101 (PMC3580493; doi:10.1186/gb-2012-13-11-r101)
Supplement: Additional file 14 — Table with qPCR primer sequences and efficiency. [file gb-2012-13-11-r101-S14.PDF]

Primer sequences and efficiency

HUGO gene name	Synonym	Forward primer 5'-3'	Reverse primer 5'-3'	Efficiency[1]
RPLP0	hARP	caccattgaaatcctgagtgatgt	tgaccagcccaaaggagaag	2.00
DEFB4	hBD-2	gatgcctcttccaggtgttttt	ggatgacatatggctccactctt	1.99
DEFB103A	hBD-3	gtgaagcctagcagctatgaggat	tgattcctccatgacctggaa	2.04
PI3	Elafin	catgagggccagcagctt	tttaacaggaactcccgtgaca	2.02
S100A7	Psoriasin	cttccttagtgcctgtgacaaaaa	aaggacagaaactcagaaaaatcaatct	1.89
S100A8	MRP8	ccgagtgtcctcagtatatcaggaa	acgcccatctttatcaccagaat	1.98
LYZ	lysozyme	ccgtgatccacaaggcatta	ggacatctctgttttgacaacgat	1.91
SLPI		ttcccctgtgaaagcttgattc	gatatcagtggtggagccaagtc	1.96
CAMP	LL37	ccaggcccacgatggat	accagcccgtccttcttga	1.85

[1]*Efficiency as fold increase in fluorescence per PCR cycle.*
