# Supplementary material for: Microbiome dynamics of human epidermis following skin barrier disruption
Source: Genome Biol. 2012 Nov 15;13(11):R101. doi: 10.1186/gb-2012-13-11-r101 (PMC3580493; doi:10.1186/gb-2012-13-11-r101)
Supplement: Additional file 2 — Microbial community composition of upper buttock skin and forehead. Relative abundances of the most abundant bacterial taxa on (A) upper buttock, and (B) forehead. The figure illustrates the average composition of the sample for different levels of bacterial taxonomy, from the domain level (left) to the genes level (right). Figure generated using software described in Sundquist et al [69], also providing or more detailed description of the visualization. [file gb-2012-13-11-r101-S2.PDF]

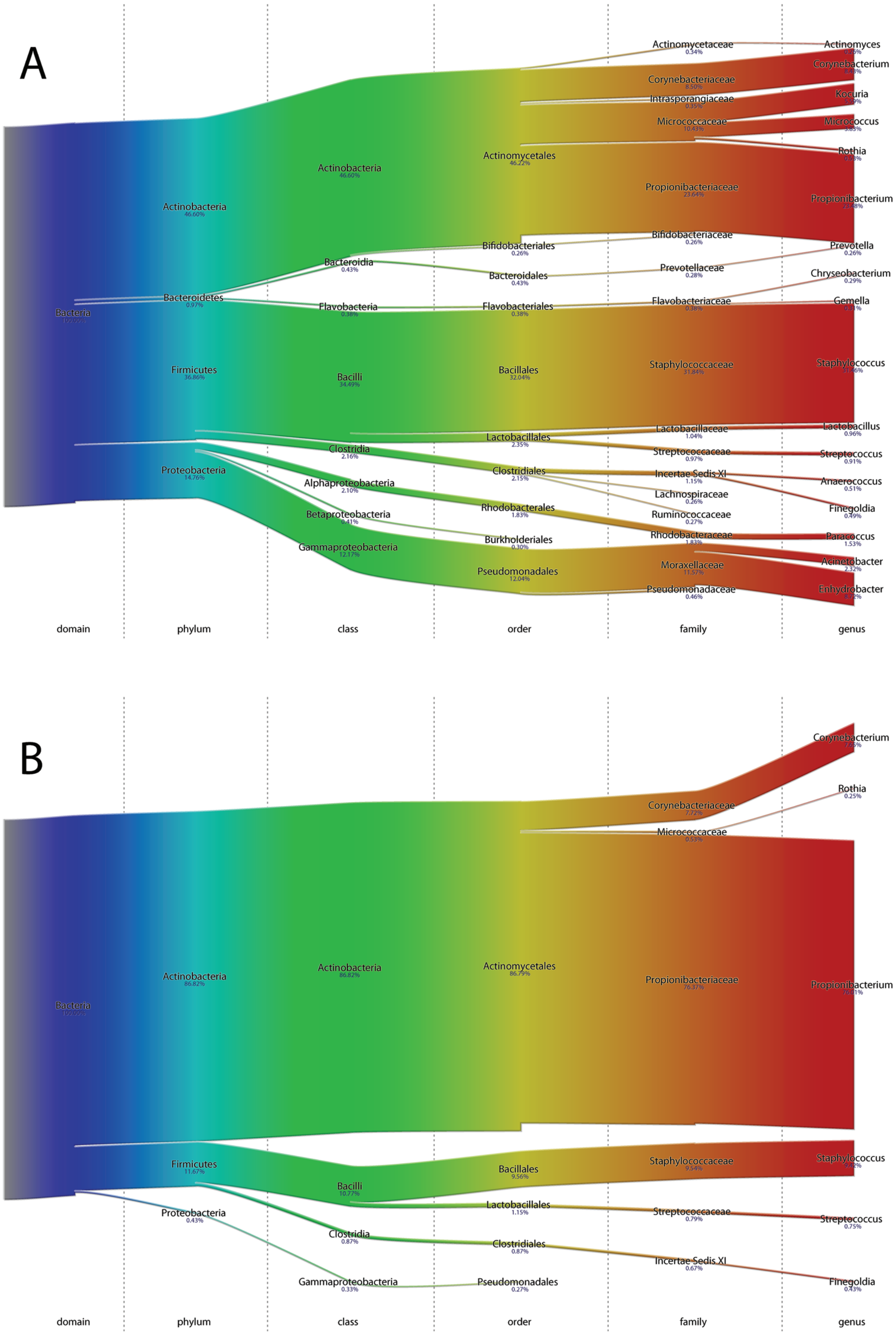
A
Bacteria
100.00%
Actinobacteria
46.60%
Bacteroidetes
0.97%
Firmicutes
36.86%
Proteobacteria
14.76%
Actinobacteria
46.60%
Bacteroidia
0.43%
Flavobacteria
0.38%
Bacilli
34.49%
Clostridia
2.16%
Alphaproteobacteria
2.10%
Betaproteobacteria
0.41%
Gammaproteobacteria
12.17%
Actinomycetales
46.22%
Bifidobacteriales
0.26%
Bacteroidales
0.43%
Flavobacteriales
0.38%
Bacillales
32.04%
Lactobacillales
2.35%
Clostridiales
2.15%
Rhodobacterales
1.83%
Burkholderiales
0.30%
Pseudomonadales
12.04%
Actinomycetaceae
0.34%
Corynebacteriaceae
8.50%
Intrasporangiaceae
0.35%
Micrococcaceae
10.43%
Propionibacteriaceae
23.64%
Bifidobacteriaceae
0.26%
Prevotellaceae
0.28%
Flavobacteriaceae
0.38%
Staphylococcaceae
31.84%
Lactobacillaceae
1.04%
Streptococcaceae
0.97%
Incertae Sedis XI
1.15%
Lachnospiraceae
0.26%
Ruminococcaceae
0.27%
Rhodobacteraceae
1.83%
Moraxellaceae
11.57%
Pseudomonadaceae
0.46%
Actinomyces
0.26%
Corynebacterium
8.43%
Kocuria
5.59%
Micrococcus
3.83%
Rothia
0.53%
Propionibacterium
23.48%
Prevotella
0.26%
Chryseobacterium
0.29%
Gemella
0.31%
Staphylococcus
31.46%
Lactobacillus
0.96%
Streptococcus
0.91%
Anaerococcus
0.51%
Finegoldia
0.49%
Paracoccus
1.53%
Acinetobacter
2.32%
Enhydrobacter
8.72%
domain
phylum
class
order
family
genus
B
Bacteria
100.00%
Actinobacteria
86.82%
Firmicutes
11.67%
Proteobacteria
0.43%
Actinobacteria
86.82%
Bacilli
10.77%
Clostridia
0.87%
Gammaproteobacteria
0.33%
Actinomycetales
86.79%
Bacillales
9.56%
Lactobacillales
1.15%
Clostridiales
0.87%
Pseudomonadales
0.27%
Corynebacteriaceae
7.72%
Micrococcaceae
0.53%
Propionibacteriaceae
76.37%
Staphylococcaceae
9.54%
Streptococcaceae
0.79%
Incertae Sedis XI
0.67%
Corynebacterium
7.65%
Rothia
0.25%
Propionibacterium
76.01%
Staphylococcus
9.42%
Streptococcus
0.75%
Finegoldia
0.43%
domain
phylum
class
order
family
genus
